# A Brief History of an Existing Controversy on the Subject of Assimilated Rank in the Navy of the United States

**Published:** 1850-11

**Authors:** 


*A brief History of an Existing Controversy on the Subject of Assimilated Rank in the Navy of the United States.* By W. S. W. R. Philadelphia. 8 vo., pp. 108.

Our readers are probably aware that an effort is being made by the medical officers of the navy to obtain an *assimilated rank* in the service. The force of this expression may not be fully understood by all. We select, therefore, in explanation, from the work, the title page of which prefixes this article, the following extract:—

*Assimilated rank* is the relative position of members of staff corps to officers of the line, and to officers of different staff-corps.

As authority to command is not inherent to rank of any description but originates exclusively from the instructions or orders of the Commander-in-chief of the army, that is, the President, assimilated rank cannot confer a right to command in the line. A colonel of engineers cannot command a major in the line, nor a medical officer, who may be a major or captain in assimilated rank.

Consuls enjoy assimilated rank as captains in the navy; but the possession of this kind of rank does not confer upon them any right to command junior captains, commanders or lieutenants; nor have those officers in the navy any right to command a consul. But it serves to regulate points of etiquette, precedence, &c., on occasions of ceremony between consuls and officers of the navy on foreign stations.

The effect of assimilated rank may require further illustration. A captain in the line commands a company, every member of which is bound to obey his orders; without such obedience, the functions of the office of captain in the line could not be performed. An assistant-surgeon, who has assimilated rank as a captain, has no authority in virtue of his assimilated rank to command in the line: he cannot act as captain and direct the duties of the company under any circumstances whatever; not being of the military profession, technically speaking, not in the line of succession in the grade of captains by the rule of seniority, or any other rule, he can never occupy a position to discharge the official duties of a captain. The right of the medical officer to command is limited to the immediate control and direction of those of the company who are in need of medical attention, and such persons are bound to obey him; and he is properly responsible for all that relates to the care and management of the sick and wounded of the company, as well as the attendants upon them. From the nature of the case, he must have this degree of authority in order to discharge the duties of medical officer, and therefore his official commission confers it upon him. His assimilated rank adds nothing to the measure of his authority, which he derives exclusively from his commission. But his assimilated rank, as a captain, entitles him, in his official and social relations with members of the military community, to receive the same signs of respect, which are conventional, as a captain in the line; and on occasions of ceremony, such as military reviews, parades, marches, funerals, &c., and also, when serving as a member of a military council, or as a member of a board of survey, or a military court, his assimilated rank entitles him to take a place among those of the grade of captains, which is determined by the dates of their several commissions as captains, the earliest date taking precedence. Assimilated rank, no matter how high a grade it may attain, does not confer authority to command those possessing *lineal* rank in any grade, not even in the lowest; but those of an *inferior lineal* rank have no authority to command those possessed of *assimilated* rank of a higher grade; that is, cadets, second lieutenants, first lieutenants of the line cannot rightfully command or take precedence of those who have assimilated rank as captains. Nor can those who have *lineal* rank as captains take precedence and command of those whose *assimilated* rank as captains is of older date, especially when in the presence of a common superior—a major of the line, for example. Hence it is that assimilated rank is a conservative, or protective rank; without it, the officers of the medical, or any staff department of an army, might be made subordinate even to privates, and find no redress in law for such indignity.

It has long been a subject of complaint with naval medical officers that, not possessing an assimilated rank, their position has been imperfectly defined, and, in consequence, the rights and privileges due to the corps are not always accorded. The end sought for is, that Congress shall take such action in the matter as shall leave no room for doubt or cavil on the part of secretaries, commanders, and subordinate officers. For this end petitions have been transmitted, not only from the medical officers of the navy, but from the National Medical Association, and numerous local societies in different parts of the Union. The nature of the application is such, that it concerns, not alone the medical branch of the navy, but the whole profession of the United States. A similar source of complaint heretofore existed in the army, but, through the persevering exertions of the present Surgeon General, an assimilated rank has at length been obtained. It is to be hoped that the efforts of our brethren in the navy will be equally successful.

The whole subject is fully discussed in the work referred to, the initials being of those of Dr. Ruschenberger, Surgeon, U. S. Navy—a name well known for many valuable contributions to medicine and natural science. The courteous tone which pervades the discussion by Dr. R., denotes the true gentleman.

